# Anti-Tumor and Radiosensitization Effects of *N*-Butylidenephthalide on Human Breast Cancer Cells

**DOI:** 10.3390/molecules23020240

**Published:** 2018-01-25

**Authors:** Yi-Ju Su, Sung-Ying Huang, Yu-Hui Ni, Kuan-Fu Liao, Sheng-Chun Chiu

**Affiliations:** 1 Department of Radiation Oncology, Taichung Tzu Chi Hospital, Buddhist Tzu Chi Medical Foundation, Taichung 42743, Taiwan; daniellesyr@gmail.com; 2 Department of Ophthalmology, Hsinchu Mackay Memorial Hospital, Hsinchu 30071, Taiwan; hopes929@gmail.com; 3 Department of Research, Taichung Tzu Chi Hospital, Buddhist Tzu Chi Medical Foundation, Taichung 42743, Taiwan; s008029@yahoo.com.tw; 4 Graduate Institute of Integrated Medicine, China Medical University, Taichung 40402, Taiwan; kuanfuliaog@gmail.com; 5 Department of Internal Medicine, Taichung Tzu Chi Hospital, Buddhist Tzu Chi Medical Foundation, Taichung 42743, Taiwan; 6 Department of Laboratory Medicine, Taichung Tzu Chi Hospital, Buddhist Tzu Chi Medical Foundation, Taichung 42743, Taiwan; 7 General Education Center, Tzu Chi University of Science and Technology, Hualien 97005, Taiwan

**Keywords:** apoptosis, breast cancer, G2/M arrest, metastasis, *N*-butylidenephthalide, radiosensitization

## Abstract

*N*-Butylidenephthalide (BP), which is extracted from a traditional Chinese medicine, *Radix Angelica Sinensis* (*danggui*), displays antitumor activity against various cancer cell lines. The purpose of this study was to investigate the cytotoxic and radiosensitizing effect of BP and the underlying mechanism of action in human breast cancer cells. BP induces apoptosis in breast cancer cells, which was revealed by the TUNEL assay; the activation of caspase-9 and PARP was detected by western blot. In addition, BP-induced G2/M arrest was examined by flow cytometry and the expression levels of the G2/M regulatory protein were detected by western blot. BP also suppresses the migration and invasion of breast cancer cells, which was tested by wound healing and the matrigel invasion assay; the involvement of EMT-related gene expressions was detected by real-time PCR. Furthermore, BP enhanced the radiosensitivity of breast cancer cells, which was measured by the colony formation assay and comet assay, where the foci of γ-H2AX after radiation significantly increased in BP pretreated cells and was evidenced by immunocytochemistry staining and western blot. The homologous recombination (HR) repair protein Rad51 was down-regulated after BP pretreatment. These results indicate that BP might be a potential chemotherapeutic and radiosensitizing agent for breast cancer therapy.

## 1. Introduction

Breast cancer is the most commonly diagnosed malignancies among women and the leading cause of cancer death in females from Western countries, with an estimated 1.7 million new breast cancer cases and 522,000 deaths worldwide in 2012 [1]. Furthermore, the incidence of breast cancer is expected to rise in the coming years and the age of patients is tending to be younger. Treatment options for breast cancer patients are limited to conventional cytotoxic chemotherapy, which is not effective against high-grade malignant tumors [2,3,4]. Radiation therapy plays a critical role in the local and regional control of epithelial carcinomas including breast cancer. Recent attempts have focused on using potential chemotherapeutic chemicals as radiosensitizing agents to enhance the efficacy of radiation therapy.

Recently, non-traditional treatments using herbs and dietary supplements have been considered as alternative medicines for cancer therapy. The naturally-occurring compound, *N*-butylidene-phthalide (BP; C_12_H_12_O_2_) extracted from *Angelica sinensis* (also called danggui in Chinese), has been reported to exhibit growth inhibitory activity on various human cancer cell lines, including brain, lung, and liver cancer cells [5,6,7]. There have been extensive investigations into the potential mechanisms of BP as an anti-tumor agent in various tumor cells. For example, BP induces mitochondria-dependent and ER stress-mediated apoptosis in prostate cancer [8,9]. BP represses the transcriptional activity of hTERT via down-regulating the expression of AP-2, leading to the telomerase activity inhibition in lung cancer [7]. BP down-regulates the expression of S-phase kinase-associated protein (Skp2), resulting in cell cycle G0/G1 phase arrest and tumor senescence in glioblastoma multiforms (GBM) [10]. BP also reduces the epithelial-mesenchymal transition (EMT) via the modulation of enhancements of Zeste 2 (EZH2) and AXL receptor tyrosine kinase (AXL) in GBM [11,12]. These findings indicate that BP is a promising anticancer compound with the potential for clinical application. However, the potentials of BP on breast cancer therapy or as radiosensitizing agent have not been addressed and require further clarification.

As cells are most radiosensitive to radiation during the cell cycle G2/M phase [13], G2/M arrest is the major cause of cell death induced by anti-tumor or radiosensitizing agents. The progression of the cell cycle from the G2 to M phase depends on the activity of the G2/M cell cycle checkpoints [14]. The checkpoint protein kinase Chk2 inhibits the activity of cdc25c via phosphorylation at ser216, which prevents the activation of cdc2, leading to the inactivation of the cyclin B-cdc2 complex. To prove this working hypothesis, we examined whether BP induced cell cycle arrest, investigated the expression of cell cycle regulatory proteins, and measured the radiosensitivity of BP-treated human breast cancer cells in this study.

In this study, we also determined the BP induced G2/M phase arrest on human breast cancer cells. BP also induced mitochondria-mediated apoptosis and inhibited metastatic activity in breast cancer cells. In addition, we demonstrated that BP could radiosensitize breast cancer cells to radiation and was effective at DNA damage induction. Accordingly, BP might be a potential anti-tumor and radiosensitizing agent for breast cancer therapy.

## 2. Results

### 2.1. Anti-Proliferation and Apoptosis Induction of BP in Breast Cancer Cells

**Figure 1:**
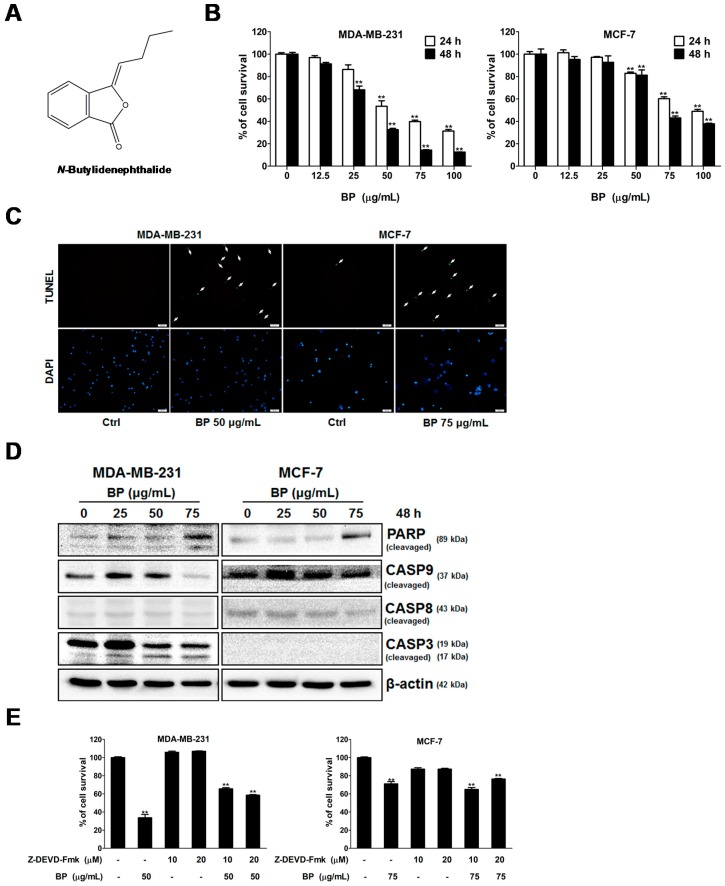
Effects of BP on the viability of human breast cancer cells. (**A**) Molecular structure of BP, C_12_H_12_O_2_, MW: 188.23; (**B**) Human breast cancer cells were treated with 0.2% DMSO as vehicle control or increasing concentration of BP (12.5 to 100 μg/mL) for 24 (□) and 48 h (■), respectively, and the survival rate was evaluated with the MTT assay; (**C**) Human breast cancer cells were treated in the presence or absence of BP for 48 h and then were fixed and stained with the TUNEL assay. Nuclei were stained with DAPI. TUNEL positive cells are indicated by arrows. Scale bar: 50 μm; Panel (**D**) Human breast cancer cells were treated with 25, 50 and 75 μg/mL BP for 48 h, and Western blot analysis was performed for cleaved PARP, caspase-9, caspase-8, and caspase-3. β-actin was used as an internal control; (**E**) Human breast cancer cells pretreated with caspase-3 inhibitor Z-DEVD-fmk (10 or 20 μM) for 1 h and then treated in the presence or absence of BP for 48 h, the survival rate was evaluated with the MTT assay. Data are presented as means ± S.D. obtained from three different experiments. ** *p* < 0.01 vs. vehicle.

For determining the effect of BP on cell viability, human MDA-MB-231 and MCF-7 breast cancer cell lines were incubated with various concentrations of BP (12.5 to 100 μg/mL) for 24 or 48 h, followed by MTT assay analysis (Figure 1B). The results showed that BP suppressed the breast cancer cells growth in a time- and dose-dependent manner, the EC_50_ values at 48 h were 46.7 μg/mL (MDA-MB-231) and 77.4 μg/mL (MCF-7). Accordingly, we used the 50 (MDA-MB-231) and 75 μg/mL (MCF-7) for further experiments. 

To elucidate the role of apoptosis in BP induced breast cancer cell death, the TUNEL assay was performed to detect apoptotic cells that undergo DNA degradation during the late stage of apoptosis. The TUNEL positive cells (green fluorescence) were significantly increased after BP treatment when compared to the control (Figure 1C). The activation of caspase family proteins form part of the critical steps for apoptosis and was also observed by western blot. BP induced cleavages of PARP, caspase-9 and -3 in a dose-dependent manner on MDA-MB-231, but not MCF-7 cells (Figure 1D). Since it has been widely reported that MCF-7 cells do not express caspase-3, treatment with BP did not affect the expression of cleaved caspase-3 in MCF-7 cells. However, MCF-7 cells are still sensitive to cell death induction by several stimuli, including staurosporine, PBOX-6, and other DNA-damaging agents [15,16]. Treatment with BP can increase the expression of PARP and caspase-9 in MCF-7 cells. The involvement of caspase-3 activation was further evidenced by the caspase-3 inhibitor Z-DEVD-fmk pretreatment in MDA-MB-231 cells, but not MCF-7 cells (Figure 1E).

Cells were pretreated with Z-DEVD-fmk (10 or 20 μM) for 1 h and then treated in the presence or absence of BP for 48 h, followed by MTT assay analysis. The caspase-3 inhibitor pretreatment partly inhibited the BP-induced cell death from 65.6% and 58.6% (MDA-MB-231) when compared to the BP alone group (33.7%). However, the caspase 3 inhibitor pretreatment did not inhibit the BP-induced cell death in MCF-7 cells (65% and 74.5%) when compared to the BP alone group (71.1%). These results suggested that BP induced breast cancer cell death via the caspase-3-dependent and caspase-3-independent pathways.

### 2.2. BP Induced Accumulation of G2/M Cell Population in Breast Cancer Cells

**Figure 2:**
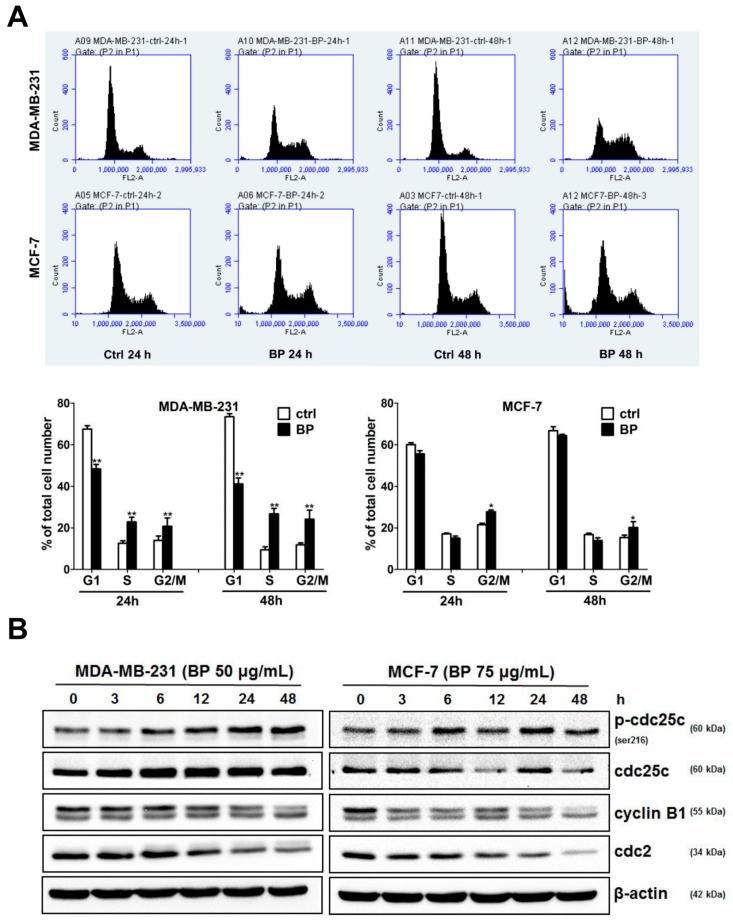
BP induced accumulation of G2/M cell population and changed the expression profiles of G2/M regulatory proteins in human breast cancer cells. (**A**) Human breast cancer cells were incubated with BP for 24 and 48 h and then analyzed by flow cytometry. Data are presented as mean ± S.D. obtained from three independent experiments, * *p* < 0.05 vs. vehicle; ** *p* < 0.01 vs. vehicle; (**B**) Human breast cells were treated with BP for 3 to 24 h. Western blot analysis of p-cdc25c (ser216), cdc25c, cyclin B1, and cdc2 was performed.

It is well known that cells at the G2/M phase are the most radiosensitive population to radiation therapy, so the stages of cell cycle affected by BP were determined. Human breast cancer cells were treated in the presence or absence of BP for 24 and 48 h followed by flow cytometry analysis (Figure 2A). 

BP induced an increase in the proportion of cells in G2/M phase (MDA-MB-231: 24.2% and MCF-7: 20.2%) after 48 h treatment in comparison with the control cells (MDA-MB-231: 11.8% and MCF-7: 15.3%, Figure 2A). We further examined the expression levels of the cell cycle regulatory proteins after BP treatment by western blot (Figure 2B). The level of p-cdc25c (ser216) was increased and the levels of cyclin B1 and cdc2 were decreased, which implicated the increasing of cell population at the G2/M phase. Together, these data suggest that BP induced accumulation of G2/M cell population in breast cancer cells and might be a potential candidate for radiosensitizing agent.

### 2.3. BP Inhibited the Migratory and Invasive Ability of Breast Cancer Cells

**Figure 3:**
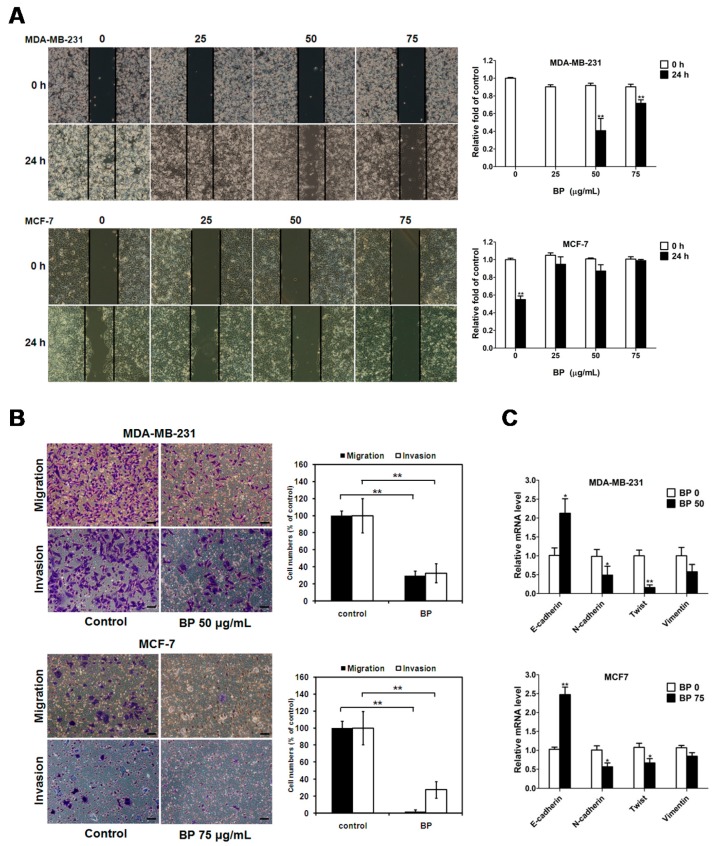
Effects of BP on the migratory and invasive ability on human breast cancer cells. (**A**) Wound healing assay: BP inhibits human breast cancer cell migration in a dose-dependent manner. Images of wound closures were captured using a microscope with ×100 magnification; scale bar: 100 μm. The cell-free area invaded by migrated cells across the black lines were calculated by three randomized fields and quantified. The cell-free distance at 0 h were set at 100%; (**B**) Human breast cancer cells were pretreated with BP for 24 h, then seeded onto the trans-well hanging insert coating with (invasion) or without (migration) matrigel for 24 h. Images were captured using an inverted microscope with ×200 magnification; scale bar: 50 μm. The migration and invasion of human breast cancer cells were quantified by enumerating the stained cells that migrated into the underside of the hanging insert membrane; (**C**) Human breast cancer cells were treated with BP for 24 h, and the mRNA expression levels of E-cadherin, N-cadherin, Twist, and vimentin were determined by qRT-PCR analysis. Data are presented as means ± S.D. from three different experiments. * *p* < 0.05 vs. vehicle; ** *p* < 0.01 vs. vehicle.

Although progress has been achieved in treating breast cancer, metastatic breast cancer remains the major challenge in breast cancer therapy. To investigate the anti-metastatic effect of BP on breast cancer cells, wound-healing and trans-well assays were performed (Figure 3). 

The results of the wound-healing assay showed that BP treatment significantly decreased cell migration in both breast cancer cell lines in a dose-dependent manner (Figure 3A). Moreover, BP significantly inhibited the migration (MDA-MB-231: 29.8% and MCF-7: 2.2%) and invasion (MDA-MB-231: 32.5% and MCF-7: 27.6%) of breast cancer cells which was revealed by the trans-well migration and matrigel-invasion assays. Epithelial-mesenchymal transition (EMT) plays a prominent role in tumor invasion and metastasis. We further examined the EMT-related gene expression after BP treatment by qRT-PCR. The results showed that BP could inhibit migration and invasion in breast cancer cells via the up-regulation of the epithelial marker E-cadherin and down-regulation of mesenchymal markers N-cadherin and vimentin, and the down-regulation of transcription factor Twist in both breast cancer cell lines (Figure 3B,C). These data suggested that BP inhibited the migratory and invasive ability of breast cancer cells. 

### 2.4. BP Pretreatment Followed by IR Reduced the Colony Forming Ability

**Figure 4:**
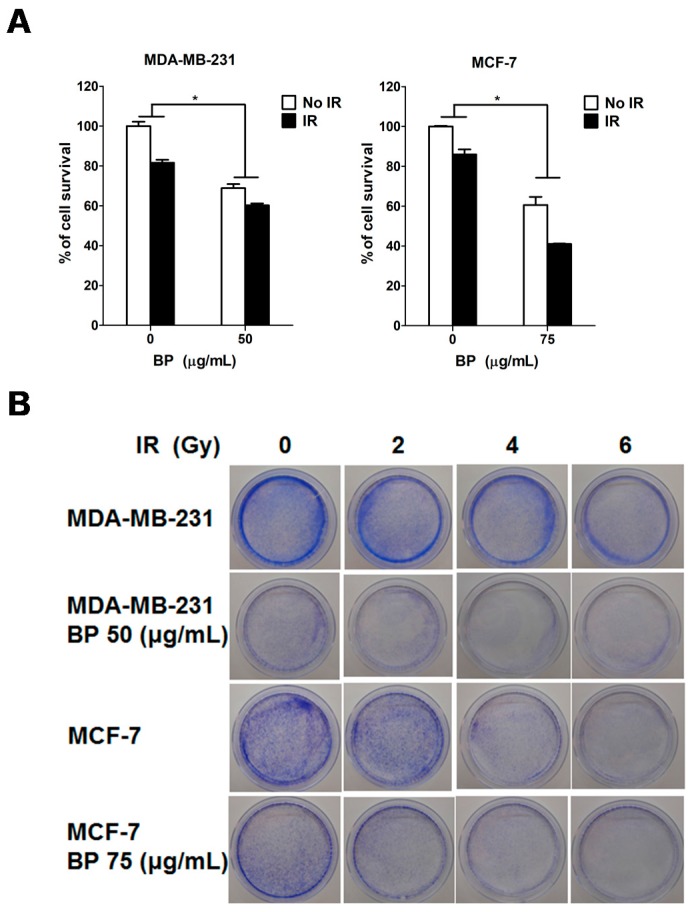

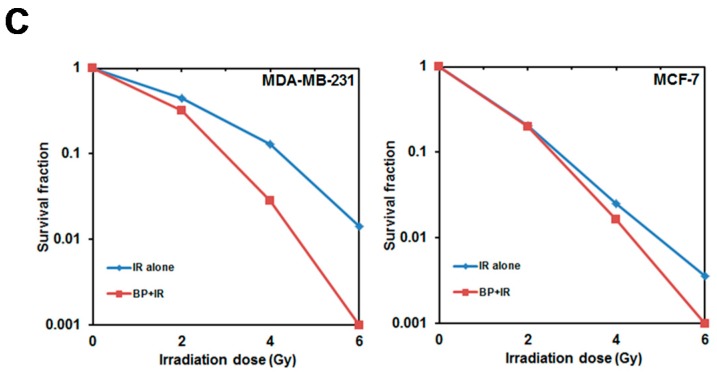
Effect of BP and/or IR treatment on the clonogenic survival of human breast cancer cells. (**A**) Human breast cancer cells were treated in the presence or absence of BP for 24 h and then exposed with or without IR (6 Gy). After 24 h, the cell survival rate was evaluated with the MTT assay; (**B**) Human breast cancer cells were treated in the presence or absence of BP for 24 h and were then exposed to IR (2 to 6 Gy). After 14 days, colonies with cells greater than 50 were stained with 2% crystal violet and counted; (**C**) The number of colonies was counted and the surviving fraction was normalized to the corresponding control by the survival curve analysis. The values are the mean ± S.D. from three independent experiments, * *p* < 0.05 vs. vehicle.

To elucidate the radiosensitizing effect of BP on human breast cancer cells, both cell lines were pre-treated with DMSO or BP for 24 h, followed by IR (6 Gy) treatment. Combining radiation with BP suppressed cell viability more effectively than either radiation or BP treatment alone (Figure 4A). In a further clonogenic assay, combination with BP pre-treatment was effective in blocking colony formation in both cell lines when compared to the IR alone group (Figure 4B). The survival fraction at 4 Gy (SF4) was used to evaluate the radiosensitivity. In the MDA-MB-231 cells, SF4 was 0.028% for cells pretreated with BP and 0.13% for the IR alone, with a significant reduction of 78.5%. In the MCF-7 cells, SF4 was 0.016% for cells pretreated with BP and 0.025% for the IR alone, with a significant reduction of 35.2% (Figure 4C). Taken together, these results suggested that BP potentiated the radiosensitivity of both breast cancer cells.

### 2.5. BP Pretreatment Followed by IR Increased IR-Induced DNA Damage and Inhibited DNA Repair

**Figure 5:**
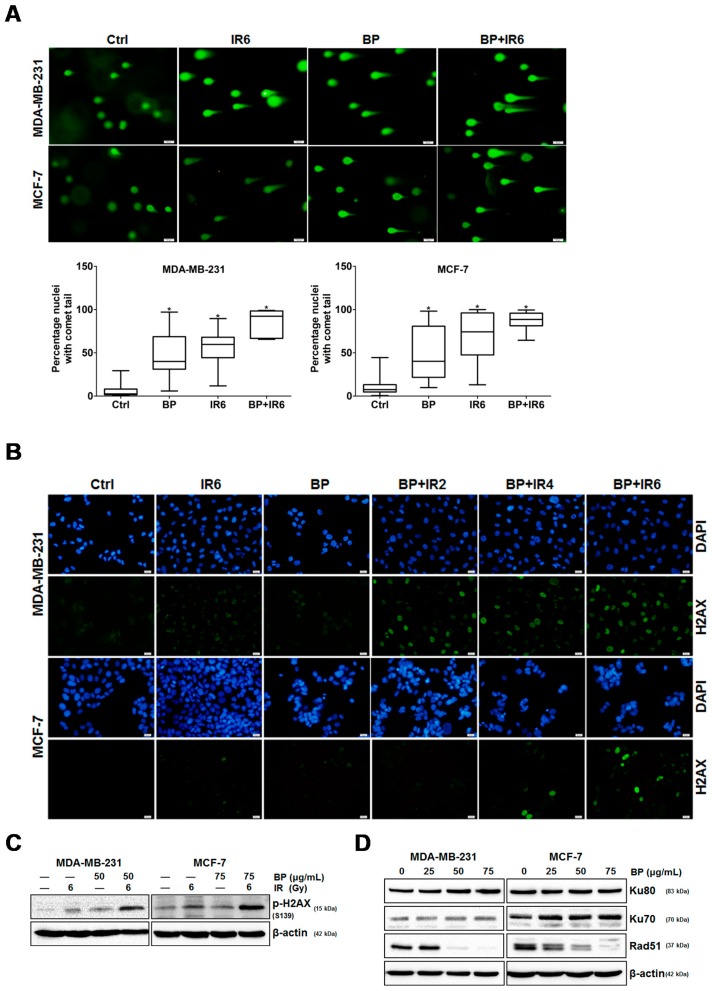
DNA damage induced by BP and/or IR treatment in human breast cancer cells. (**A**) Human breast cancer cells were treated in the presence or absence of BP for 24 h and then exposed with or without IR (6 Gy). After 48 h, the cells were harvested and the comet assay performed. The percentage of DNA in the tail was plotted as determined by OpenComet. The values are the mean ± S.D. from three independent experiments, * *p* < 0.05 vs. vehicle; (**B**) Human breast cancer cells were treated in the presence or absence of BP for 24 h, exposed with IR (2, 4 or 6 Gy), and then fixed for immunofluorescence analysis of the γ-H2AX foci. Nuclear staining was done with DAPI (blue) while γ-H2AX staining appeared green; (**C**) The cells were pre-treated in the presence or absence of BP for 24 h and then exposed with or without IR (6 Gy). The cell lysates were harvested and analyzed for p-H2AX (ser139) by western blot; (**D**) Human breast cancer cells were treated with 25, 50 and 75 μg/mL BP for 48 h, and western blot analysis was performed for Ku80, Ku70, and Rad51.

Increasing the induction of DNA damage by IR is considered to be one of the most important abilities of radiosensitizers [17,18]. An increase in DNA double-strand break (DSB) has been shown to be related to the synergic effect between IR and radiosensitizers. Therefore, we examined whether BP could induce radiosensitization on human breast cancer cells through an increase in DNA damage. The comet assay is a sensitive method for detecting DSB from an individual cell [19]. Cells containing damaged DNA have the appearance of a comet, and the degree of DNA damage can be measured by tail length and the percentage of DNA in the tail. Human breast cancer cells were pre-treated in the presence or absence of BP for 24 h then with or without IR (6 Gy) treatment. The comet assay was performed 48 h after IR treatment. As shown in Figure 5A, the combined treatment of BP and IR greatly increased the comet tail length when compared with cells treated with IR or BP alone. The percentage of DNA in the tail was plotted and scored as determined by OpenComet.

The phosphorylation on Ser139 of γ-H2AX is one of the key events of DNA damage response and considered as a marker of DSB [20]. We further investigated the involvement of DNA damage in the BP-induced radiosensitization by detecting the formation of γ-H2AX. Cells were pretreated in the presence or absence of BP for 24 h and followed with or without exposure to IR. The γ-H2AX foci were significantly increased in the BP-IR co-treatment group when compared to the IR alone group detected by immune-cytochemistry staining (Figure 5B). The expression level of phosphorylated H2AX (γ-H2AX) was increased in the BP-IR co-treatment group when compared to the BP or IR alone group evidenced by western blot (Figure 5C). These results suggested that BP pre-treatment radiosensitized the breast cancer cells via the impairment of the repair of IR-induced DSBs.

DSBs have two major repair pathways: homologous recombination (HR) and non-homologous DNA end-joining (NHEJ). Rad51 is a central player in HR, whereas Ku70 and Ku80 are essential for NHEJ. We therefore examined the expression of Ku70, Ku80, and Rad51 by western blot to assess whether these two pathways were altered in BP-treatment breast cancer cells. As shown in Figure 5D, there were no obvious changes in the levels of Ku80 and Ku70 after BP treatment. However, BP significantly inhibited the expression levels of Rad51 in a dose-dependent manner in both breast cancer cell lines. These results suggested that BP sensitized the cell to IR-induced DNA damage, and the disturbance of DSB repair by BP might be the major cause impairing DNA repair in cells at the G2/M phase.

## 3. Discussion

BP is a naturally-occurring compound that exhibits anti-proliferation and apoptosis-induction activity in various cancer cells [5,6,7,8,9]. However, the potentials of BP on breast cancer therapy have not been determined. This study aimed to explore the anticancer activity of BP on breast cancer by using the MCF-7 and MDA-MB-231 human breast cancer cell lines. Breast cancer can be divided into distinct subtypes that have prognostic and therapeutic implications. MCF-7 cells are estrogen receptor (ER)-positive, progesterone receptor (PR)-positive, and HER2 (human epidermal growth factor receptor 2)/neu negative. MDA-MB-231 cells are ER-negative, PR-negative, and HER2/neu negative, the so-called triple-negative breast cancer (TNBC). Interestingly, both cell lines showed proliferation inhibition to BP treatment in a time- and dose-dependent manner, especially the MDA-MB-231 cells. This result implied that the response of MCF-7 and MDA-MB-231 to BP was independent from ER or PR status. The results of the TUNEL assay implicated the involvement of apoptosis in BP-induced growth inhibition on breast cancer cells. Moreover, the relationship between the activation of individual caspases in these two breast cancer cells was of particular interest. BP-induced apoptosis through the activation of caspase-3 and can be partly rescued by caspase-3 inhibitor pretreatment in MDA-MB-231 cells. Furthermore, a previous study reported that MCF-7 cells lack caspase-3, which is caused by a 47-base pair deletion within exon 3 of the *CASP-3* gene that resulted in completely abrogating the translation of the CASP-3 mRNA [21]. In addition, a previous report indicated that although MCF-7 cells do not express the caspase-3 protein, it can still undergo apoptosis accompanied by DNA fragmentation via the activation of caspase-7 [15,21,22]. The involvement of caspase-7 in BP-induced apoptosis in MCF-7 cells should be further investigated.

Treatment options for breast cancer therapy are limited to cytotoxic chemotherapy, which is not effective against high-grade tumors. Thus, the combination of radiation therapy with radiosensitizing agents is one of the most effective treatments against breast cancer. It is well known that the cancer cells are more radiosensitive in the cell cycle G2/M phase. The compound which can arrest the cell cycle at the G2/M phase may function as a radiosensitizer. We further examined whether BP induced breast cancer cell cycle arrested at the G2/M phase. BP induced accumulation of G2/M cell population in both breast cancer cell lines after 48 h treatment. In addition, BP increased the expression level of p-cdc25c (ser216) and decreased the expression levels of cyclin B1 and cdc2. Thus, the increasing of G2/M cell population induced by BP might enhance radiosensitivity in human breast cancer cell lines. 

Clonogenic cell death, resulting from the loss of reproductive integrity, is a critical property of cell death induced by IR treatment. Therefore, measuring the clonogenic ability is the major end-point in radiobiology by clonogenic assay, and plays a pivotal role in studying IR effects. Despite the relatively high dose (6 Gy) used in the radiosensitization experiment in Figure 4A (MTT assay, 24 h), the BP pre-treatment sensitized both breast cancer cell lines to IR with the lower dose (2 or 4 Gy) after 14 days in clonogenic assay (Figure 4B,C). These results implicated that BP might be a potent radiosensitizer candidate. 

An increase in DNA DSBs and an impaired DNA repair system have been shown to be related to the ability of potential radiosensitizers. The combination treatment of BP and IR significantly increased the DNA DSBs when compared with the BP or IR alone group, which was evidenced by the comet assay (Figure 5A,B). The phosphorylated form of H2AX, which is termed γ-H2AX, is a sensitive indicator of DNA DSB and corresponds to DNA repair in cells that are exposed to IR. The expression level of γ-H2AX was significantly enhanced following the combination treatment of BP and IR when compared with BP or IR alone (Figure 5C). These results indicated that there were increased DNA DSBs after BP-pretreatment in breast cancer cells. There are two major DSB repair pathways in mammalian cells: homologous recombination (HR) and non-homologous DNA end-joining (NHEJ). Rad51 is a crucial player for the former, whereas ku80 and ku70 are essential for the latter. As shown by western blot analysis, the expression level of Rad51 decreased dose-dependently in both cell lines after BP treatment. However, the expression levels of Ku80 and Ku70 did not change significantly when compared to the control cells after BP treatment. A previous report indicated that the efficiency of HR was significantly elevated in breast cancer cell lines, suggesting that the inhibition of HR might have a selective effect against breast cancer [23]. 

Although progress has been achieved in treating breast cancer patients, approximately 90% of breast cancer deaths are caused by local invasion or the distant metastasis of tumor cells. Epithelial-mesenchymal transition (EMT) is a phenotypic conversion linked with metastasis. In the EMT processes, cell loss of epithelial properties occurs with the down-regulated expression of epithelial markers such as E-cadherin. At the same time, they gain mesenchymal properties and increased motility with the up-regulated expression of mesenchymal markers such as N-cadherin and vimentin. In the present study, we demonstrated that BP significantly inhibited the migration and invasion of human breast cancer cells in the wound-healing and trans-well assays. Furthermore, BP could inhibit the process of EMT with an increased level of the epithelial marker E-cadherin and a decreased level of mesenchymal markers (N-cadherin and vimentin), and transcription factor Twist. Together, these findings indicated that BP inhibited EMT in human breast cancer cells.

The clinical application of BP is limited due to several problems. First, the structure of BP is light and thermal labile [24,25]. Second, the effective concentrations of BP are relative higher than other drugs such as paclitaxel and doxorubicin on breast cancer therapy in vitro. In addition, the water insolubility of BP might lead to low bioavailability in vivo. Therefore, the development of drug delivery system that can protect the structure of BP or increase its aqueous solubility is needed. Recent studies demonstrated the better efficacy of BP after encapsulated with lipo-PEG-PEI complex (LPPC) or biodegradable interstitial release polymer in vitro and in vivo which providing better strategies for use in breast cancer therapy [12,24]. 

This study demonstrated that BP had anti-proliferation and induced accumulation of G2/M cell population in human breast cancer cells. BP also induced apoptosis via the mitochondrial-mediated pathway in human breast cancer cells. The anti-metastatic effect of BP on human breast cancer cells was shown by the wound healing and trans-well assays. Furthermore, we demonstrated for the first time that BP could radiosensitize human breast cancer cells to radiation by increasing DNA damage, and the down-regulation of the homologous recombination repair protein, Rad51. These results suggested that BP may be a potential therapeutic agent and a potent radiosensitizer for breast cancer therapy.

## 4. Materials and Methods

### 4.1. Chemicals and Antibodies

BP (C_12_H_12_O_2_, 95%) was purchased from Alfa Aesar (Ward Hill, NY, USA). Dimethyl sulfoxide (DMSO), crystal violet, [3-(4,5-dimethylthizol-2-yl)-2,5-diphenyltetrazolium bromide] (MTT), Tween-20, methanol, and horseradish peroxidase-conjugated secondary antibodies were purchased from Sigma Chemical Co. (St. Louis, MO, USA). The antibodies were all purchased from Cell Signaling Technology, Inc., (Danvers, MA, USA). Polyvinyldenefluoride (PVDF) membranes, BSA protein assay kit and chemiluminescence reagents were purchased from Amersham Biosciences (Arlington Heights, IL, USA).

### 4.2. Cell Culture

The human breast cancer cell lines MDA-MB-231 and MCF-7 were purchased from the Bioresource Collection and Research Center (BCRC, Hsinchu, Taiwan), and cultured in its standard medium as recommended by the BCRC. Briefly, MDA-MB-231 was cultured in Leibovitz’s L-15 medium with 2 mM l-glutamine and 10% fetal bovine serum (FBS) at 37 °C in a humidified atmosphere without CO_2_. MCF-7 was cultured in a minimum essential medium Eagle with 0.1 mM non-essential amino acids, 1 mM sodium pyruvate and 10% FBS at 37 °C in a humidified atmosphere with 5% CO_2_. Culture medium, FBS, and cultured supplements were all purchased from Invitrogen (Carlsbad, CA, USA). Cell lines were authenticated annually by short-tandem repeat analysis and routinely tested for mycoplasma contamination (BCRC).

### 4.3. MTT Assay

The viability of the cells following treatment with various BP dosages was evaluated using the MTT assay preformed in triplicate. Briefly, cells (4 × 10^4^/well) were incubated in 24-well plates containing 0.5 mL of serum-containing medium. Cells were allowed to adhere for 18–24 h and were washed with phosphate-buffered saline (PBS). Solutions were always freshly prepared by dissolving 0.1% DMSO (control) or BP in the culture medium before being added to cells. The BP-containing medium was removed after treatment for 24 or 48 h, as indicated. The cells were then washed with PBS, replenished with culture medium containing 300 μg/mL MTT, and incubated for 1 h at 37 °C. After the MTT medium was removed, 0.5 mL of DMSO was added to each well. Absorbance at 570 nm was detected by a multi well plate reader Infinite 200 Pro Tecan^TM^ (Tecan, Mannedorf, Switzerland). The absorbance for DMSO-treated cells was considered as 100%.

### 4.4. Cell Cycle Analysis

The cell cycle was determined by flow cytometry following DNA staining to reveal the total amount of DNA. Approximately 5 × 10^5^ of breast cancer cells were incubated with 50 (MDA-MB-231) or 75 (MCF-7) μg/mL BP for the indicated time. Cells were harvested with trypsin/EDTA, collected, washed with PBS, fixed with cold 70% ethanol overnight, and then stained with a solution containing 20 μg/mL PI, 0.2 mg/mL RNase A, and 0.1% Triton X-100 for 30 min in the dark. The cells were then passed through an Accuri C6 flow cytometer (BD Biosciences, San Jose, CA, USA) to measure the DNA content. The data were obtained and analyzed with CFlow^®^ software (v1.0.264.21, BD Biosciences, San Jose, CA, USA).

### 4.5. Western Blot Analysis

5 × 10^5^ cells per 6-cm petri dish were lysed with 200 μL M-PER mammalian protein extraction reagent containing a protease inhibitor cocktail (Thermo Scientific, Rockford, IL, USA) and centrifuged at 13,000× *g* at 4 °C for 10 min. The protein concentration in the supernatants was quantified using a BSA Protein Assay Kit. Electrophoresis was performed on a 12% SDS-PAGE gel in a Mini-PROTEAN Tetra cell electrophoresis System (BioRad, Hercules, CA, USA) using 20 μg of protein extract for each lane. Resolved proteins were transferred to PVDF membranes, blocked with 5% skim milk for 1 h at room temperature, and finally probed with the specific primary antibodies at 4 °C overnight. The PVDF membrane was washed three times with TBS/0.2% Tween-20 at room temperature and then incubated with the appropriate secondary antibody labeled with horseradish peroxidase (goat anti-mouse or anti-rabbit, 1:10,000, Sigma Chemical) for 1 h at room temperature. All resolved protein bands were detected using Western Lightning Chemiluminescence Reagent Plus (Amersham Biosciences).

### 4.6. Cell Migration and Invasion Assay

Cell migration was determined by the wound healing and the trans-well assays. For the wound healing assay, cells were seeded and grown overnight to 90~95% confluence in 24-well plates. Migration was tested in the wound-healing assays using culture inserts (ibidi, Martinsried, Germany). Cells were washed with PBS and cultured with medium containing 0–75 μg/mL BP. Wound closure was evaluated and photographed at 24 h with an inverted microscope (CKX41 fluorescence microscope, Olympus, Melville, NY, USA). 

Cell migration was determined by the trans-well assays performed using hanging inserts (Millipore Co., Billerica, MA, USA) in a 24-well plate. Cells were seeded (5 × 10^4^) in the hanging inserts, which was then filled with culture medium or with medium supplemented with 50 (MDA-MB-231) or 75 (MCF-7) μg/mL BP. Culture medium supplemented with 10% FBS was added to the bottom chamber. Incubation was carried out at 37 °C for 24 h. The hanging inserts were washed with PBS, and cells on the upper filter surface were wiped away with a cotton swab. The inserts were subsequently fixed with 10% formalin for 10 min at room temperature, stained with 0.2% *w*/*v* crystal violet, and washed with PBS; the remaining cells were counted on the opposite side of the filter under a light microscope (Olympus CKX41 fluorescence microscope) at ×200 magnification. The migration cell numbers of the control group were considered as 100%. For the invasion assay, a matrigel basement membrane matrix (BD Biosciences, San Jose, CA, USA) was coated to the upper side of the hanging inserts at a concentration of 2 mg/mL. Cells were seeded onto the coated hanging inserts and followed by the migration assay protocol. 

### 4.7. RNA Extraction and Real-Time RT-PCR Analysis

Total RNA was extracted from cell lines using an-RNeasy Mini Kit^®^ (Qiagen, Valencia, CA, USA) and reverse transcribed at 37 °C for 60 min with an Omniscript RT Kit^®^ (Qiagen) according to the manufacturer’s instructions. Real-time RT-PCR analysis was performed in triplicate in a Step One Plus Real-Time PCR system (Applied Biosystems, Foster City, CA, USA) with Power SYBR^®^ Green PCR Master Mix (Applied Biosystems) in a final volume of 20 μL/reaction. The threshold cycle (*C*_t_) value of each tested gene was normalized to the C_t_ value of the GAPDH control from the same RNA preparation. The ratio of transcription of each gene was calculated as 2^−(Δ*C*t)^, where Δ*C*_t_ is the difference *C*_t(test gene)_ − *C*_t(GAPDH)_. The real-time RT-PCR primer sequences used in this study were: N-cadherin F-5′-ACAGTGGCCACCTACAAAGG-3′, R-5′-CCGAGATGGGGTTGATAATG-3′, E-cadherin F-5′-ACGTCGTAATCACCACACTGA-3′, R-5′-TTCGTCACTGCTACGTGTAGAA-3′, Twist F-5′-CGGGAGTCCGCAGTCTTA-3′, R-5′-TGAATCTTGCTCAGCTTGTC-3′, vimentin F-5′-TCTAC GAGGAGGAGATGCGG-3′, R-5′-GGTCAAGACGTGCCAGAGAC-3′, and GAPDH F-5′-CCATGG AGAAGGCTGGGG-3′, R-5′-CAAAGTTGTCATGGATGACC-3′. 

### 4.8. Radiation Exposure

Human breast cancer cells were cultured in a 25 T flask with either the vehicle control (DMSO) or BP for 24 h. After 24 h, cells were irradiated at room temperature using a linear accelerator (Varian Trilogy Linear Accelerator, Varian Medical Systems, Palo Alto, CA, USA) at a dose rate of 3 Gy/min. After IR treatment, cells were kept at 37 °C in an incubator for subsequent experiments.

### 4.9. Clonogenic Assay

Approximately 10^5^ of cells were plated in 10-cm culture dishes and incubated overnight to allow for recovery from trypsinization and the resumption of cell cycle progression. Next, cells were pre-treated with either the vehicle control (DMSO) or BP for 24 h and then irradiated using the dosages of 2 or 4 Gy. The cells were then returned to the incubator undisturbed for colony formation. After 14 days, colonies (containing ≥50 cells) were stained with 2% crystal violet in methanol for 30 min. The number of colonies was then counted and normalized with the corresponding control. The plating efficiency (PE) is the ratio of the number of colonies to the number of cells seeded in the non-irradiated group. The survival fraction (SF) was calculated as the mean number of colonies/(cells seeded × PE). In the radiation survival curve analysis, the different conditions were normalized to the control. 

### 4.10. Comet Assay

A DNA damage assay was carried out using an OxiSelect Comet Assay kit (STA-351, Cell Biolabs, San Diego, CA, USA). Briefly, cells were seeded in a 6-well plate, treated with or without BP (MDA-MB-231: 50 μg/mL, MCF-7: 75 μg/mL) for 24 h, and then exposed to 6 Gy IR. After 48 h, the cells were harvested and the comet assay performed according to the manufacturer’s instructions. The comet tails were imaged using an Olympus CKX41 fluorescence microscope at ×400 magnification. The images were analyzed using OpenComet software (v1.3.1, OpenComet is an open-source software tool providing automated analysis of comet assay images. The OpenComet plug-in and source code is available at www.opencomet.org.) [26].

### 4.11. Immunofluorescence Staining for γ-H2AX

Cells were plated at a density of 4 × 10^4^ cells/well in a 24-well plate covered by a coverslip and allowed to attach for 24 h. Next, the cells were treated with BP (MDA-MB-231: 50 μg/mL, MCF-7: 75 μg/mL) for 24 h followed by 2, 4, or 6 Gy radiation. After 24 h post-IR, the cells were fixed with cold 4% paraformaldehyde for 10 min. The cells were washed twice in PBS, and incubated in cold permeabilization solution (0.3% Triton X-100 + 0.1% sodium citrate) for 10 min and subsequently blocked with 5% BSA at room temperature for 1 h. The cells were then incubated with an anti γ-H2AX (Cell Signaling) antibody at 4 °C overnight. The cells were washed with PBST three times and then incubated with FITC-conjugated secondary antibody for 1 h at room temperature. The cells were then washed with PBS three times and stained with 300 nM DAPI for 10 min. Images were obtained using an Olympus CKX41 fluorescence microscope at ×400 magnification. 

### 4.12. Statistical Analysis

All data are shown as mean ± S.D. Statistical differences were analyzed using the Student’s *t*-test for normally distributed values and by nonparametric Mann–Whitney *U*-test for values with a non-normal distribution. Significant differences between groups were evaluated using analysis of variance (ANOVA) with Games–Howell test as a post-hoc test. All the values for graph in this study from three independent experiments.

## Abbreviations

BP	*N*-butylidenephthalide
DSB	DNA double-strand break
EMT	epithelial-mesenchymal transition
HR	homologous recombination
NHEJ	non-homologous DNA end-joining
